# Dietary patterns related to biological mechanisms and survival after breast cancer diagnosis: results from a cohort study

**DOI:** 10.1038/s41416-023-02169-2

**Published:** 2023-02-03

**Authors:** Carlota Castro-Espin, Catalina Bonet, Marta Crous-Bou, Verena Katzke, Charlotte Le Cornet, Franziska Jannasch, Matthias B. Schulze, Anja Olsen, Anne Tjønneland, Christina C. Dahm, Christian S. Antoniussen, Maria Jose Sánchez, Pilar Amiano, María Dolores Chirlaque, Marcela Guevara, Claudia Agnoli, Rosario Tumino, Carlotta Sacerdote, Maria Santucci De Magistris, Malin Sund, Stina Bodén, Torill Enget Jensen, Karina Standahl Olsen, Guri Skeie, Marc J. Gunter, Sabina Rinaldi, Esther M. Gonzalez-Gil, Elisabete Weiderpass, Sofia Christakoudi, Alicia K. Heath, Laure Dossus, Antonio Agudo

**Affiliations:** 1 Unit of Nutrition and Cancer, Catalan Institute of Oncology-ICO, L’Hospitalet de Llobregat, Barcelona, Spain; 2 Nutrition and Cancer Group, Epidemiology, Public Health, Cancer Prevention and Palliative Care Program, Bellvitge Biomedical Research Institute-IDIBELL, L’Hospitalet de Llobregat, Barcelona, Spain; 3 Department of Epidemiology, Harvard T.H. Chan School of Public Health, Boston, MA 02115 USA; 4 German Cancer Research Center (DKFZ), Heidelberg, Germany; 5 German Institute of Human Nutrition Potsdam-Rehbruecke, Dept. of Molecular Epidemiology, Arthur-Scheunert-Allee, Nuthetal, Germany; 6 Institute of Nutritional Science, University of Potsdam, Nuthetal, Germany; 7 Danish Cancer Society Research Center, Diet, Cancer and Health, Copenhagen, Denmark; 8 Department of Public Health, University of Copenhagen, Copenhagen, Denmark; 9 Department of Public Health, Aarhus University, Aarhus, Denmark; 10 Escuela Andaluza de Salud Pública (EASP), Granada, Spain; 11 Instituto de Investigación Biosanitaria ibs.GRANADA, Granada, Spain; 12 Centro de Investigación Biomédica en Red de Epidemiología y Salud Pública (CIBERESP), Madrid, Spain; 13 Department of Preventive Medicine and Public Health, University of Granada, Granada, Spain; 14 Ministry of Health of the Basque Government, Sub Directorate for Public Health and Addictions of Gipuzkoa, San Sebastian, Spain; 15 Biodonostia Health Research Institute, Epidemiology of Chronic and Communicable Diseases Group, San Sebastián, Spain; 16 Department of Epidemiology, Regional Health Council, IMIB-Arrixaca, Murcia University, Murcia, Spain; 17 Navarra Public Health Institute, Pamplona, Spain; 18 Navarra Institute for Health Research (IdiSNA), Pamplona, Spain; 19 Epidemiology and Prevention Unit, Department of Research, Fondazione IRCCS Istituto Nazionale dei Tumori, Milan, Italy; 20 Hyblean Association for Epidemiological Research, AIRE ONLUS Ragusa, Ragusa, Italy; 21 Unit of Cancer Epidemiology, Città della Salute e della Scienza University-Hospital, Turin, Italy; 22 A.O.U. Federico II, Naples, Italy; 23 Department of Surgical and Perioperative Sciences/ Surgery, Umeå University, Umeå, Sweden; 24 Department of Surgery, University of Helsinki and Helsinki University Hospital, Helsinki, Finland; 25 Department of Clinical Sciences - Pediatrics, Umeå University, Umeå, Sweden; 26 Department of Community Medicine, UiT the Arctic University of Norway, Tromsø, Norway; 27 Nutrition and Metabolism Branch, International Agency for Research on Cancer (IARC-WHO), Lyon, France; 28 Department of Epidemiology and Biostatistics, School of Public Health, Imperial College London, London, UK; 29 Department of Inflammation Biology, School of Immunology and Microbial Sciences, King’s College London, London, UK

**Keywords:** Cancer epidemiology, Breast cancer, Epidemiology, Nutrition

## Abstract

**Background:**

Inflammatory, insulin and oestrogenic pathways have been linked to breast cancer (BC). We aimed to examine the relationship between pre-diagnostic dietary patterns related to these mechanisms and BC survival.

**Methods:**

The diabetes risk reduction diet (DRRD), inflammatory score of diet (ISD) and oestrogen-related dietary pattern (ERDP) were calculated using dietary data from the European Prospective Investigation into Cancer and Nutrition (EPIC) study. Cox proportional hazards models were used to assess associations between dietary patterns and overall mortality and competing risk models for associations with BC-specific mortality.

**Results:**

We included 13,270 BC cases with a mean follow-up after diagnosis of 8.6 years, representing 2340 total deaths, including 1475 BC deaths. Higher adherence to the DRRD score was associated with lower overall mortality (HR_1–SD_ 0.92; 95%CI 0.87–0.96). Greater adherence to pro-inflammatory diets was borderline associated with 6% higher mortality HR_1–SD_ 1.06; 95%CI 1.00–1.12. No significant association with the oestrogen-related dietary pattern was observed. None of the dietary patterns were associated with BC-specific mortality.

**Conclusions:**

Greater adherence to an anti-diabetic and anti-inflammatory diet prior to diagnosis is associated with lower overall mortality among BC survivors. Long-term adherence to these dietary patterns could be a means to improve the prognosis of BC survivors.

## Introduction

Breast cancer (BC) accounts for one in four cancer cases and one in six cancer deaths among women worldwide [1]. Declines in breast cancer mortality rates have been reported in many high-income countries, likely due to the combined effects of earlier detection by screening and improvements in treatment. As a result, 5-year survival after breast cancer diagnosis has increased steadily, reaching 90% in North American countries, Australia, New Zealand, and in many European countries, although with large differences across countries and world regions [2]. Despite the relatively high survival rates, large differences are still observed across age and stage at diagnosis [2, 3]. Therefore, for the effective management of BC, there is a need to investigate modifiable factors that could impact long-term prognosis.

A comprehensive review of the literature concluded that the current evidence suggests that excess body fatness is a predictor of poor survival, while physical activity may be associated with better prognosis among BC survivors [4]. The same review reported that there is evidence of links between better survival after BC diagnosis and some foods or dietary components. However, the evidence is not strong enough to make specific recommendations. Most previous research looked at individual foods, food groups or individual nutrients. More recently, dietary patterns have been used to better capture the complexity of dietary intake in contrast to single foods or nutrients. Accordingly, several epidemiological studies have investigated the possible role of diet through dietary patterns in BC survivors [5].

Prospective observational studies have found that dietary patterns defined as “healthy” or based on dietary guidelines (e.g., the Healthy Eating Index) are associated with better survival among breast cancer survivors [6–8]. Although the current trend of recommending cancer survivors to follow cancer prevention guidelines seems prudent, having specific recommendations for breast cancer survivors could have an important impact on prognosis. In relation to this, a promising approach is the study of dietary patterns based on the underlying biological processes or mechanisms of the relationship between diet and breast cancer prognosis [9].

Breast cancer is strongly influenced by hormones, especially oestrogens, which are involved in the aetiology of this disease [10]. High levels of endogenous sex hormones, especially oestrogens, are associated with postmenopausal breast cancer risk and current research suggests that diet influences endogenous hormone levels [11]. In addition, insulin resistance and hyperinsulinaemia, as well as chronic low-grade inflammation, are associated with obesity, which is a strong predictor of mortality, and sedentary behaviours, which are both linked to breast cancer risk and prognosis [12, 13]. Moreover, breast cancer survivors with type 2 diabetes (T2D) seem to have poor breast cancer prognosis, including increased risk of recurrence [14], and some of the mechanisms that may influence the neoplastic process include hyperinsulinemia and chronic inflammation [15–17]. A few dietary patterns have recently emerged in nutritional research aimed at assessing the biological mechanisms underlying associations between diet and breast cancer [18–20].

Herein, we examined the associations between adherence to three previously developed dietary patterns—the diabetes risk reduction diet (DRRD), the inflammatory score of diet (ISD), and the oestrogen-related dietary pattern (ERDP)—and breast cancer-specific and all-cause mortality among breast cancer survivors identified in the European Prospective Investigation into Cancer and Nutrition (EPIC) study. We hypothesised that greater adherence to a low-risk diet for T2D, an anti-inflammatory diet, and a more anti-oestrogenic diet, may be associated with better prognosis outcomes in breast cancer survivors.

## Methods

### Study population and case ascertainment

We used data from EPIC, a collaborative cohort study of more than half a million middle-aged adults recruited between 1992 and 2000. The methods have been previously described in further detail [21]. Briefly, at recruitment participants completed questionnaires on diet, lifestyle, and medical history, anthropometric parameters were measured, and a blood sample was drawn. All participants provided informed consent and EPIC was approved by the Ethics Committee of the International Agency for Research on Cancer (IARC), Lyon, France, as well as the local ethics committees of the study centres.

After excluding participants with prevalent tumours at recruitment, subjects without information on follow-up, lifestyle, and diet, as well those with implausible diet (extreme values of ratio between energy intake and energy requirement), a total of 318,686 women from nine countries participated in this study (Denmark, France, Germany, Italy, The Netherlands, Norway, Spain, Sweden, and the United Kingdom).

Incident breast cancer cases and vital status were identified through population-based cancer registries in Denmark, Italy, the Netherlands, Spain, Sweden, and the United Kingdom. In France and Germany, a combination of follow-up methods was used including health insurance records, cancer pathology registries and active follow-up of study participants and their next of kin. Follow-up for cancer endpoints, vital status, and causes of death was available until 2015.

We used the International Classification of Diseases for Oncology (ICD-O-2) and breast cancer coded as C50-50.9 to define breast cancer cases. A total of 13,320 incident primary malignant breast cancer cases were identified; after excluding 7 cases with unknown vital status, 12 with inconsistent follow-up data, and 31 with non-epithelial morphology, a total of 13,270 breast cancer cases (of which 14 in situ) were included in the present analysis. During the follow-up 2,340 cases died, of which 1475 due to breast cancer. Specific causes of death of women included in this analysis are shown in Table S1. Available information on the tumour receptor status of breast cancer cases, obtained from pathology reports, is generally limited varying by country and receptor type (Table S2).

### Dietary assessment and derivation of dietary patterns

Lifestyle data collected at recruitment included questions on education, occupation, history of previous illness, lifetime history of smoking habit and alcohol consumption, and physical activity level. Usual diet during the previous 12 months was assessed at recruitment using validated country/centre-specific dietary questionnaires, mainly food frequency questionnaires and, to a lesser extent, diet history questionnaires [21, 22]. The participants’ nutrient intakes were calculated by multiplying the daily amount consumed of each food item by its nutrient content using country-specific food composition tables [23].

The three dietary patterns used have been previously described in detail [18, 24, 25]. The DRRD is an a priori defined dietary pattern and the ISD and ERDP are data-driven. The three indices include food groups, individual foods or nutrients and use two different scoring systems. Intakes of the dietary components used in the DRRD and ERDP were expressed as food or nutrient density per 2000 kcal (intake in g/day, divided by the subject’s energy intake and multiplied by 2000 kcal). On the other hand, energy is one of the components in the ISD.

The DRRD, developed by Rhee et al. [24] and further adapted by Kang et al. [19], includes 9 dietary components (Table S3), scored between one and five according to the distribution in quintiles of the intake. This value is assigned in ascending order for components whose consumption is consistent with low risk of T2D (cereal fibre, coffee (caffeinated and decaffeinated), nuts, ratio of polyunsaturated-saturated fats, whole fruits), and in descending order for components associated with high T2D risk (glycemic index, trans-fat, sugar-sweetened beverages and fruit juices, and red and processed meat). Thus, the DRRD score ranges from 9 (lowest adherence) to 45 (highest adherence), with higher scores indicating lower risk of T2D.

The ISD was initially based on the Dietary Inflammatory Index (DII) [26]. A modified version of the original ISD that includes 27 dietary components and excludes alcohol is used in this study (Table S4) [20]. Detailed methods to calculate the ISD have been described elsewhere [25]. Briefly, each component of the ISD, mainly nutrients, is assigned an inflammatory weight based on its association with six known inflammatory biomarkers (interleukin-1b (IL-1b), IL-4, IL-6, IL-10, tumour necrosis factor alpha (TNFα) and C-reactive protein (CRP)) [26]. The intake of each component is standardised using the mean and standard deviation (SD) from the EPIC population. The z scores were converted to percentile scores and then centred on 0 (by doubling each percentile score and subtracting 1); these values were then multiplied by the respective inflammatory weight to obtain the food item-specific ISD, which were summed to produce the overall ISD for each participant. The value of the ISD for an individual is a relative index that allows the diets of individuals to be categorised on a continuum from maximally anti-inflammatory to maximally pro-inflammatory. Adherence to the ISD resulted in a score between –4.7 (lowest adherence) and 5.4 (highest adherence).

Similarly, the ERDP score is comprised of foods with assigned weights according to levels of unconjugated oestradiol and the ratio of 2- and 16-hydroxylated oestrogen metabolites [18]. To calculate the ERDP in the present study we used the weights as reported in reference [18] for a total of 11 food items included in the original pattern and available in the EPIC databases (Table S5). Positively weighted intakes were assigned to refined grains, tomatoes, cruciferous vegetables, cheese, fish/shellfish high in ω-3 fatty acids, processed meats (frankfurters/luncheon meats in the original ERDP) and negatively weighted intakes were assigned to nuts/seeds, other vegetables, fish/shellfish low in ω-3 fatty acids, yogurt, and coffee. Adherence to the ERDP score ranged from –1.8 (lowest adherence) and 1.2 (highest adherence).

### Statistical analysis

We used Cox proportional hazards models to prospectively analyse the association between the exposures (dietary patterns) and overall mortality. Fine-Grey competing risks models [27], considering other causes of death as a competing event, were applied for breast cancer-specific mortality. Entry time was considered as the date of diagnosis of primary breast cancer, and exit time was the date of death or end of follow-up.

All survival models were stratified by country and menopausal status at diagnosis (women aged ≥55 years at diagnosis were considered postmenopausal regardless of the baseline information). Multivariable-adjustment for potential confounders included age at diagnosis (5-years categories), attained level of education (none, primary school, secondary school, technical/professional school, longer education, unknown), body mass index (underweight, normal-weight, overweight, obese), physical activity (inactive, moderately inactive, moderately active, active, unknown), alcohol consumption reported at recruitment (non-drinker, >0–3, >3–12, >12–24,>24 g/day, unknown), smoking habits and intensity as cigarettes per day (cig/day) at recruitment (never, current 1–15 cig/day, current 16–25 cig/day, current >25 cig/day, former quit ≤10 years before recruitment, former quit 11–20 years before recruitment, former quit more than 20 years before recruitment, current smoker of cigars, pipes and occasional current smokers, current smokers with missing information on the intensity, unknown), ever use of hormone replacement therapy for menopause at diagnosis (yes, no, unknown), tumour stage (0/I, II, III, IV, non-metastatic but unknown specific stage, unknown), tumour grade (well-differentiated, moderately differentiated, poorly differentiated or undifferentiated, not determined), and tumour receptor status (positive, negative, unknown) for: oestrogen receptor (ER), progesterone receptor (PR), and human epidermal growth factor receptor 2 (HER2).

The proportional hazards assumption was evaluated checking the graphs of the scaled Schoenfeld residuals. To further assess the association of mortality outcomes with dietary patterns on a continuous scale, restricted cubic spline models were used (Fig. S1), and non-linearity was tested using the Likelihood (LR) ratio test. Moreover, mutually adjusted models were performed for the three dietary patterns after testing for interaction between dietary patterns using pairwise interaction terms.

We fitted different models as follows: first, each dietary pattern was introduced independently as a categorical variable in quartiles, with the first quartile as the reference category. Tests for linear trend were performed using median value for each quartile of dietary patterns. Second, dietary patterns were used as continuous variables by 1–SD increase in the scores. On the other hand, mutually adjusted models were fitted by including the three dietary patterns as quartiles. The scores of the three dietary patterns were correlated; the Pearson correlation coefficients of DRRD with ISD and ERDP were –0.36 and –0.23, respectively, and the coefficient between ISD and ERDP was 0.12. Therefore, the residuals for each dietary pattern from a separate multiple regression model for each, including the other two patterns, were used as continuous variables to assess the association of mortality with a 1 SD increase in score.

Furthermore, we also dichotomised DRRD and ISD (below or equal to and above the median) and assessed the cross-classification of these patterns according to low-low, high-high, high-low DRRD and ISD categories, respectively, compared to the reference category (low-DRRD, high-ISD) in relation to overall mortality. We assumed that the mechanisms by which a low score of DRRD is associated with high risk of T2D is insulin resistance (IR). We used the label high-IR for women with DRRD below the median, and low-IR for those with DRRD score equal or above the median. Direct-adjusted survival curves by DRRD-ISD categories were derived from the multivariable Cox model with country and menopausal status as adjustment variables, followed by the rest of the covariates [28].

Subgroup analyses by menopausal status, body mass index (BMI), physical activity, stage of tumour, and hormone receptor status, were performed for DRRD, ISD and ERDP scores (mutually adjusted models). Heterogeneity across groups was determined using the LR test.

Sensitivity analyses were performed by additionally adjusting the survival models for time from recruitment to diagnosis, to check the assumption of stability of dietary assessment, and for the period of diagnosis to check potential influence of improvements in treatment and diagnosis over time. Stratified analyses were explored by categories of these two variables: time from diet measurement to diagnosis (<5 years, 5 to <8 years, 8 to <12 years, ≥12 years); period of diagnosis (before 2000, between 2000 to <2004, between 2004 to <2008, 2008 onwards). The cut-off points for both variables were based on the distribution of quartiles 1, 2 and 3 of breast cancer cases. Additionally, multivariable models further adjusted by these two variables (time from diet measurement to diagnosis as continuous; period of diagnosis as categorical) were performed. An additional adjustment was made for the presence of co-morbidities (separate variables: reported cardiovascular (CVD) problem and diabetes) given the outcome is overall survival. Other models excluding diabetic BC survivors to assess the effect of the low-insulin diet in women without pre-existing influence on this pathway were performed. We finally excluded breast cancer survivors with unknown tumour stage and unknown status of HER2 receptor, in separate models, to test whether treatment differences associated with these characteristics might modify the magnitude of the associations.

## Results

Our study included 13,270 incident breast cancer cases with a mean follow-up of 8.6 years from diagnosis (SD 4.9 years), of whom 2340 died and 1475 due to breast cancer. Breast cancer cases were predominantly 55–65 years old, non-smokers, moderate alcohol consumers, in the normal-weight range (mean BMI 24.9) and physically inactive. The majority were postmenopausal (77%) and had non-metastatic tumours (82%) (Table 1).

**Table 1:** Baseline and tumour characteristics of breast cancer survivors in the EPIC cohort.

		All breast cancer survivors *N* = 13,270	
		*n*	%
Age at diagnosis (years)	<50	1342	10.1
	50–<55	2049	15.4
	55–<60	2671	20.1
	60–<65	2878	21.7
	65–<70	2332	17.6
	≥70	1998	15.1
Educational level	None/primary	3281	24.7
	Technical/professional	3035	22.9
	Secondary	3200	24.1
	Longer education	3143	23.7
	Unknown	611	4.6
Smoking status and intensity (cig/day or years)	Never	5940	44.8
	Current, 1–15 cig/day	1593	12.0
	Current, 16–25 cig/day	749	5.6
	Current, 26+ cig/day	134	1.0
	Former, quit ≤10 years	1063	8.0
	Former, quit 11–20 years	977	7.4
	Former, quit 20+ years	1099	8.3
	Miscellaneous^a^	1487	11.2
	Unknown	228	1.7
Alcohol consumption g/day	Non-drinker	1774	13.4
	>0–3	3820	28.8
	>3–12	4066	30.6
	>12–24	2069	15.6
	>24	1541	11.6
BMI (kg/m^2^)	Underweight <18	206	1.6
	Normal-weight 18 to <25	7612	57.4
	Overweight 25 to <30	3943	29.7
	Obesity ≥30	1509	11.4
Physical activity	Inactive	2673	20.1
	Moderate inactive	4720	35.6
	Moderate active	3606	27.2
	Active	2076	15.6
	Unknown	195	1.5
Menopausal status at diagnosis	Premenopausal	3070	23.1
	Postmenopausal	10,200	76.9
Ever use of menopause hormone replacement treatment	No	7487	56.4
	Yes	5323	40.1
	Unknown	460	3.5
Grade of tumour	Well-differentiated	1298	9.8
	Moderately differentiated	2917	22.0
	Poorly diff/Undiff	2503	18.9
	Not determined	6552	49.4
Stage of tumour	Stage 0/I	1954	14.7
	Stage II	1593	12.0
	Stage III	303	2.3
	Non-metastatic unk. stage	3984	30.0
	Stage IV	1777	13.4
	Unknown	3659	27.6
ER status	Negative	1678	12.6
	Positive	7500	56.5
	Unknown	4092	30.8
PR status	Negative	2612	19.7
	Positive	5072	38.2
	Unknown	5586	42.1
HER2 status	Negative	3587	27
	Positive	856	6.5
	Unknown	8827	66.5

*BMI* body mass index, *cig* cigarette, *diff* differentiated, *Undiff* undifferentiated, *ER* oestrogen receptor, *PR* progesterone receptor, *HER2* human epidermal growth factor receptor 2.

^a^Current smoker of cigars, pipes and occasional current smokers, current smokers with missing information on the intensity.

Results from the time-to-event analyses are shown in Table 2. Breast cancer survivors in the fourth quartile of the DRRD score had a 20% lower risk of overall mortality compared to the first quartile (reference), and a 7% decrease for each SD increase in the score. The ISD was borderline associated with an increased risk of overall mortality when assessed continuously: HR_SD_ 1.06; 95% CI 1.00, 1.12. Mutually adjusted models including the scores as residuals showed higher association for DRRD_Q4vsQ1_ HR 0.78 95% CI 0.68, 0.90; *p*-trend <0.001 and HR_SD_ 0.92 (0.87–0.96), remaining the same for ISD. The ERDP score, however, was not associated with survival outcomes. The assessment of associations of dietary patterns and overall mortality by means of restricted cubic splines (Fig. S1) showed no statistically significant deviation from linearity. For breast cancer-specific mortality, no associations were observed with any of the three dietary patterns, either independently or mutually adjusted (Table 2).

**Table 2:** Associations between dietary patterns and all-cause and breast cancer-specific mortality.

		HR (95% CI)				*p-* trend	HR (95% CI)
All-cause mortality		Q1	Q2	Q3	Q4		Continuous (1–SD increase)
DRRD	Multivariable model^a^	1 (ref.)	0.96 (0.86– 1.06)	0.89 (0.79– 0.99)	0.80 (0.70– 0.91)	<0.001	0.93 (0.89–0.97)
	Dietary patterns mutually adjusted^b^	1 (ref.)	0.95 (0.85– 1.06)	0.88 (0.78– 0.99)	0.78 (0.68–0.90)	<0.001	0.92 (0.87– 0.96)
ISD	Multivariable model	1 (ref.)	1.03 (0.91– 1.17)	1.12 (0.98– 1.27)	1.12 (0.96– 1.30)	0.079	1.06 (1.00–1.12)
	Dietary patterns mutually adjusted	1 (ref.)	1.01 (0.89– 1.15)	1.07 (0.94–1.22)	1.05 (0.90– 1.22)	0.423	1.06 (1.00– 1.12)
ERDP	Multivariable model	1 (ref.)	1.03 (0.91– 1.16)	0.95 (0.83–1.08)	0.99 (0.86– 1.15)	0.609	0.99 (0.94– 1.05)
	Dietary patterns mutually adjusted	1 (ref.)	0.99 (0.88– 1.12)	0.89 (0.78–1.01)	0.92 (0.79– 1.06)	0.100	0.97 (0.92– 1.03)
Breast cancer-specific mortality							
DRRD	Multivariable model	1 (ref.)	0.99 (0.87– 1.14)	0.96 (0.83– 1.11)	0.89 (0.76– 1.05)	0.167	0.96 (0.91–1.02)
	Dietary patterns mutually adjusted	1 (ref.)	0.98 (0.86–1.13)	0.94 (0.81–1.10)	0.87 (0.73–1.04)	0.135	0.96 (0.90– 1.01)
ISD	Multivariable model	1 (ref.)	1.03 (0.88– 1.20)	1.10 (0.94– 1.30)	1.12 (0.93– 1.35)	0.161	1.02 (0.96–1.10)
	Dietary patterns mutually adjusted	1 (ref.)	1.01 (0.86– 1.19)	1.08 (0.91– 1.27)	1.09 (0.90–1.32)	0.302	1.02 (0.95–1.10)
ERDP	Multivariable model	1 (ref.)	0.94 (0.81 –1.09)	0.90 (0.76– 1.06)	0.90 (0.75–1.07)	0.192	0.96 (0.90– 1.03)
	Dietary patterns mutually adjusted	1 (ref.)	0.92 (0.79– 1.08)	0.86 (0.73– 1.02)	0.86 (0.71– 1.03)	0.070	0.95 (0.89– 1.02)

All models were stratified by country and menopausal status at diagnosis and adjusted for age at diagnosis, attained level of education, physical activity, body mass index, alcohol consumption reported at recruitment, smoking habit and intensity as cigarettes per day at recruitment, ever use of hormone for menopause at diagnosis, cancer stage at diagnosis, cancer grade, and tumour receptor status: ER, PR, HER2.

*Ref.* Reference, *HR* hazard ratio, *CI* confidence interval, *DRRD* diabetes risk reduction diet score, *ISD* inflammatory score of diet, *ERDP* oestrogen-related dietary pattern, *Q* quartile.

^a^Multivariable model: Hazard ratios derived from a multivariable Cox regression model where exposure is a dietary pattern without including the other two patterns in the model.

^b^Dietary patterns mutually adjusted: hazard ratios derived from a multivariable Cox regression model where each dietary pattern is further adjusted for the other two dietary patterns.

To further explore the observed association between DRRD and overall mortality, we performed models using different versions of the DRRD without a food item each time to see if there were any components driving the effect (Table S6). The HRs remained stable without pointing to any specific component leading the association.

Associations between DRRD and ISD and overall mortality (as continuous variables, adjusted for each other) were more apparent for postmenopausal, physically inactive women, with metastatic tumours, and in those with PR+, ER+ and HER2- (with DRRD), and ER- tumours (with ISD) (Table 3). Women presenting overweight or obesity, an important predictor of breast cancer prognosis, did not show a stronger effect on the association with dietary patterns. Instead, it was women of normal-weight who appeared to show a more evident effect (DRRD_SD_ = HR 0.91(0.85–0.97; ISD_SD_ = 1.10 (1.01–1.20), presumably because it was not masked by obesity. However, none of the interaction terms were statistically significant, suggesting that there is no strong evidence for differential effects of these dietary patterns on mortality in subgroups of breast cancer survivors according to potential determinants of prognosis or features of the tumour.

**Table 3:** Associations between DRRD, ISD and ERDP mutually adjusted and overall mortality by subgroups of BC survivors.

		HR (95%CI)		
	*N* cases (events)	DRRD^a^	ISD^a^	ERDP^a^
All breast cancer survivors	13,270 (2340)	0.92 (0.87–0.96)	1.06 (1.00–1.12)	0.97 (0.92–1.03)
Menopausal status at diagnosis				
Premenopausal	3070 (527)	0.97 (0.87–1.07)	0.95 (0.83–1.08)	0.94 (0.83–1.06)
Postmenopausal	10,200 (1813)	0.91 (0.86–0.95)	1.09 (1.02–1.16)	0.99 (0.93–1.05)
* P-*value for heterogeneity^b^		0.951	0.074	0.693
BMI				
Normal-weight	7612 (1193)	0.91 (0.85–0.97)	1.10 (1.01–1.20)	1.04 (0.96–1.12)
Overweight- obesity	5452 (1113)	0.93 (0.87–1.00)	1.03 (0.95–1.12)	0.93 (0.86–1.01)
* P*-value for heterogeneity^b^		0.950	0.773	0.104
Physical activity level				
Inactive	7393 (1468)	0.90 (0.85–0.96)	1.07 (0.99– 1.16)	1.00 (0.93–1.07)
Active	5682 (837)	0.94 (0.87–1.01)	1.02 (0.92–1.12)	0.93 (0.85–1.02)
* P*-value for heterogeneity^b^		0.716	0.970	0.464
Stage of tumour				
Metastatic (stage IV)	1777 (585)	0.89 (0.81–0.99)	1.17 (1.04–1.33)	1.02 (0.91–1.14)
Non-metastatic	7834 (968)	0.95 (0.88–1.02)	1.02 (0.93–1.12)	0.99 (0.91–1.08)
* P*-value for heterogeneity^b^		0.261	0.795	0.089
Non-metastatic tumours				
Stage I^c^	1940 (108)	0.93 (0.74–1.18)	1.13 (0.85–1.50)	1.14 (0.87–1.50)
Stage II	1593 (250)	0.95 (0.81–1.12)	0.99 (0.82–1.20)	0.90 (0.76–1.07)
Stage III	303 (79)	1.17 (0.84–1.63)	0.82 (0.56–1.19)	0.93 (0.65–1.34)
* P-*value for heterogeneity^b^		0.269	0.716	0.131
Oestrogen receptor status				
ER(+)	7500 (1071)	0.91 (0.85–0.97)	1.04 (0.95–1.13)	1.01 (0.93–1.09)
ER(–)	1678 (426)	0.93 (0.84–1.05)	1.17 (1.01–1.34)	1.04 (0.91–1.19)
* P*-value for heterogeneity^b^		0.764	0.402	0.244
Progesterone receptor status				
PR(+)	5072 (620)	0.88 (0.80–0.96)	1.14 (1.02–1.29)	0.98 (0.88–1.09)
PR(-)	2612 (515)	0.92 (0.83–1.03)	1.08 (0.95–1.24)	1.07 (0.95–1.20)
* P*-value for heterogeneity^b^		0.819	0.169	0.980
HER2 status				
HER2(+)	856 (166)	1.07 (0.87–1.31)	0.99 (0.78–1.25)	1.09 (0.86–1.36)
HER2(–)	3587 (473)	0.88 (0.79–0.98)	1.12 (0.98– 1.28)	0.99 (0.87–1.12)
* P*-value for heterogeneity^b^		0.956	0.631	0.934

All models were stratified by country and menopausal status at diagnosis and adjusted for age at diagnosis, attained level of education, physical activity, body mass index, alcohol consumption reported at recruitment, smoking habit and intensity as cigarettes per day at recruitment, ever use of hormone for menopause at diagnosis, cancer stage at diagnosis, cancer grade, and tumour receptor status: ER, PR, HER2.

*BMI* body mass index, *HER2* human epidermal receptor status 2, HR, Hazard Ratio; CI, Confidence Interval; DRRD, Diabetes risk reduction diet; ISD, Inflammatory score of diet; ERDP, Oestrogen-related dietary pattern.

^a^Using the scores as residuals of multiple regression model including DRRD, ISD and ERDP.

^b^*P-*values for heterogeneity by introducing interaction terms in the multivariable models between the dietary pattern and the variable containing the subgroups using likelihood ratio tests.

^c^In situ BC cases are not included in these analyses since they are only 14 with 1 single event (death).

**Fig. 1:**
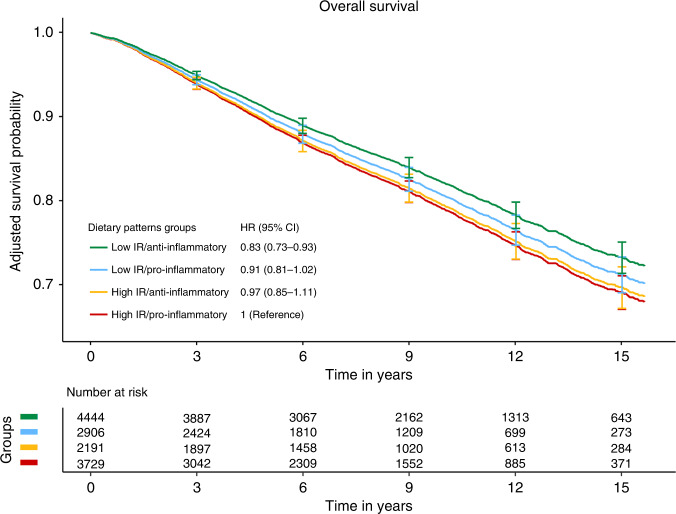
Adjusted survival curves for the four groups of the cross-classification of DRRD and ISD in relation to overall mortality in breast cancer survivors. HR hazard ratio, CI confidence interval, DRRD diabetes risk reduction diet, ISD inflammatory score of diet, IR insulin resistance. Combined variable of DRRD and ISD (dichotomised above and below median) creating a 4-level variable (low IR-anti-inflammatory, low IR-pro-inflammatory, high IR-anti-inflammatory, high IR-pro-inflammatory. HRs and CI 95% from multivariable Cox models stratified by country and menopausal status at diagnosis and adjusted for age at diagnosis, attained level of education, physical activity, body mass index, alcohol consumption reported at recruitment, smoking habit and intensity as cigarettes per day at recruitment, ever use of hormone for menopause at diagnosis, cancer stage at diagnosis, cancer grade, and tumour receptor status: ER, PR, HER2.

Since DRRD and ISD appeared to show independent effects on overall mortality, a dichotomised version of each score (above and below median) was computed to assess their combined effect (Fig. 1). Compared to women with high-IR and a pro-inflammatory diet (reference categories), higher adherence to a low-IR diet together with an anti-inflammatory diet was associated with a 17% lower risk of overall mortality (HR 0.83; 95% CI: 0.73–0.93). Intermediate diets (high-IR and anti-inflammatory diet, or low-IR and pro-inflammatory diet) showed better survival than the reference (high-IR and pro-inflammatory), but the associations did not reach statistical significance. There was no statistically significant interaction between the two dichotomised variables (DRRD and ISD scores). The adjusted survival curves for overall mortality by the four groups based on the combination of the two scores (DRRD and ISD) were consistent with the findings from the multivariable Cox model. The adjusted 5-year survival (and 95% CI) for women with high-IR and pro-inflammatory diet was 89% (88–90%) and 91% (90–92%) for those with low-IR and anti-inflammatory diet. The corresponding values for the 15-year survival were 69% (67–71%) and 73% (71–75%), respectively.

Sensitivity analyses were performed to assess the potential influence of two aspects: a possible modification of diet between the time of recruitment and diagnosis, and the period time of diagnosis (i.e., more recently diagnosed women may have received better treatments). Overall, the main results of DRRD and ISD scores, which were inversely and positively associated with all-cause mortality, respectively, remained stable (Table 4). On the other hand, analyses excluding BC survivors with diabetes or CVD reported at recruitment showed a slightly higher effect with DRRD (10% lower risk of overall mortality) compared to results including all survivors (8%). Further exclusions of breast cancer survivors with unknown tumour stage and unknown HER2 receptor status showed no attenuation of the previously observed association between DRRD and ISD and overall mortality. Finally, additional adjustment for the presence of co-morbidities in the multivariable models did not introduce any changes to the results (Table 4).

**Table 4:** Sensitivity analyses: mutually adjusted models with the three dietary patterns and overall mortality.

		HR (95% CI)^a^		
*N* (events)	N cases (events)	DRRD	ISD	ERDP
Multivariable model^b^	13,270 (2340)	0.92 (0.87–0.96)	1.06 (1.00–1.12)	0.97 (0.92–1.03)
Time from diet measurement to diagnosis				
<5 years	3842 (1086)	0.92 (0.86–0.98)	1.00 (0.92–1.1)	0.96 (0.89–1.04)
5 to <8 years	2712 (528)	0.92 (0.83–1.01)	1.05 (0.92–1.2)	0.96 (0.85–1.09)
8 to <12 years	3671 (489)	0.92 (0.83–1.02)	1.13 (0.99–1.28)	1.00 (0.89–1.14)
12 years or more	3045 (237)	0.85 (0.73–0.99)	1.14 (0.94–1.38)	1.06 (0.88–1.26)
Period of diagnosis (year)				
Before 2000	3491 (987)	0.92 (0.85–0.99)	1.02 (0.93–1.12)	0.98 (0.90–1.07)
2001–2003	3512 (680)	0.90 (0.83–0.98)	1.03 (0.93–1.15)	0.89 (0.80–0.99)
2004–2007	3470 (459)	0.93 (0.84–1.04)	1.07 (0.93–1.23)	1.04 (0.91–1.18)
2008 or later	2797 (214)	0.87 (0.75–1.02)	1.18 (0.97–1.44)	1.06 (0.88–1.29)
Excluding BC survivors				
With T2D or CVD	10,697 (1758)	0.90 (0.86–0.95)	1.06 (0.99–1.14)	1.02 (0.95–1.08)
With unknown stage of tumour	5627 (1023)	0.92 (0.86–0.99)	1.10 (1.00–1.20)	0.98 (0.90–1.06)
With unknown HER2 status	4443 (639)	0.89 (0.81–0.98)	1.10 (0.98–1.23)	1.00 (0.90–1.12)
Additional adjustments^c^				
By time from diet measurement to diagnosis (continuous)	13,270 (2340)	0.91 (0.87–0.96)	1.05 (0.99–1.12)	0.98 (0.92–1.03)
By period of diagnosis (categorical)	13,270 (2340)	0.92 (0.87–0.96)	1.05 (0.99–1.12)	0.98 (0.93–1.03)
By co-morbidities^d^	13,270 (2340)	0.91 (0.87–0.96)	1.06 (1.00–1.13)	0.97 (0.92–1.02)

^a^All models are mutually adjusted with DRRD, ISD and ERDP by adding the residuals of the two remaining scores from the multiple regression models.

^b^Model from Table 2: stratified by country and menopausal status at diagnosis and adjusted for age at diagnosis, attained level of education, physical activity, body mass index, alcohol consumption reported at recruitment, smoking habit and intensity as cigarettes per day at recruitment, ever use of hormone for menopause at diagnosis, cancer stage at diagnosis, cancer grade, and tumour receptor status: ER, PR, HER2.

^c^New variables added in the multivariable model.

^d^Including 2 co-morbidities: diabetes and a cardiovascular problem reported at recruitment.

## Discussion

In this large, prospective cohort study of 13,270 breast cancer survivors followed for a mean of 8.6 years after diagnosis, we found that women with a higher adherence to a low-risk diet for T2D (higher DRRD score) before diagnosis had lower risk of all-cause mortality after BC diagnosis. In the opposite direction, a more pro-inflammatory diet (higher ISD) was positively associated, though borderline statistically significant, with risk of all-cause mortality. The ERDP, a dietary pattern capturing the oestrogenic potential of diet, showed no association with mortality among breast cancer survivors. None of the three dietary patterns were associated with breast cancer-specific mortality. To our knowledge, this is the first time that insulinemic, inflammatory and oestrogenic potential has been assessed through dietary patterns in relation to breast cancer survival.

In line with previous excellent research on the subject, the role of diet as a protective factor in cancer survival appears to be still limited. Obesity is a strong predictor of prognosis; in fact, in our study, the HR (95%) of mortality for obesity was 1.29 (1.14–1.47). One may think that part of the relationship between diet and mortality could be mediated by BMI. However, we have accounted for the effect of obesity by including BMI in all models. Furthermore, the potential confounding effect of BMI on the associations between the anti-diabetic and anti-inflammatory patterns and mortality seem to be small: when BMI is taken out from the model the HRs (95% CI for 1–SD) increase from 0.92 (0.87–0.96) to 0.91 (0.87–0.95) for the DRRD, and from 1.06 (1.00–1.12) to 1.07 (1.01–1.14) for the ISD.

These scores were chosen because they are related to underlying biological processes or mechanisms that have been found to be associated with breast cancer risk or progression. T2D is linked to insulin resistance, which has been shown to have a negative impact on breast cancer prognosis [14, 29]. Insulin levels increase insulin-like growth factor-1 (IGF-1) activity, important in tumour initiation and progression [30], and is associated with increased oestrogen bioavailability, which promotes breast carcinogenesis. Moreover, higher dietary glycemic index, one of the components of the DRRD (and also linked to IR), has been reported to be associated with increased overall mortality [31]. In addition, metformin, a widely used treatment for patients with T2D, has been associated with lower breast cancer-specific mortality through mechanisms that induce a reduction in glucose and insulin levels [32]. This suggests that it is biologically plausible to hypothesise that increased adherence to a diet associated with a lower risk of T2D [24] may be a potential strategy to improve breast cancer prognosis. A similar conclusion was reached in the analysis of two prospective cohorts in the US [15], where greater adherence to DRRD after breast cancer diagnosis was associated with lower all-cause and breast cancer-specific mortality in a smaller sample of long-term breast cancer survivors. On the other hand, a recent study [31] reported a suggestive increased risk of CVD mortality among women with higher dietary glycemic index and glycemic load after diagnosis. Furthermore, breast cancer survivors are at increased risk of CVD owing to side effects of adjuvant breast cancer treatment. However, the limited number of deaths by CVD in our data set (Table S1) precluded exploring in deep the association of the dietary patterns of interest with CVD-related mortality.

Another mechanism that is believed to contribute to BC progression is chronic low-grade inflammation [33]. The positive association found between ISD and overall mortality (6% higher risk per 1–SD increase) is in line with this biological plausibility. Dietary components that constitute to the ISD are associated with well-known inflammatory biomarkers, including IL-1, IL-6, TNFα and CRP [25, 26]. Previous studies have reported inverse associations between healthy dietary patterns and inflammatory cytokines, but positive with Western dietary patterns [34, 35]. To the best of our knowledge, only a few studies have prospectively investigated the association between inflammatory potential of diet and survival in breast cancer patients [36, 37]. In line with our results, one of these cohort studies found that DII was positively associated with higher overall mortality and recurrence among BC survivors [36], while in the other study [37] the association was limited to cardiovascular mortality. However, these studies had a smaller number of survivors and assessed the inflammatory potential of diet using the DII, whereas we used the ISD; in the latter, intakes were standardised using the mean and SD of the EPIC population instead of a global regional database [26].

According to our results, it appears that dietary patterns related to mechanisms of insulin resistance and inflammation may have a more evident effect in postmenopausal, normal-weight, physically inactive women. This could be partially explained by the potential effects of a low-insulin resistance diet and an anti-inflammatory diet becoming evident only among women for whom hormonal pathways are less relevant and without other strong determinants of these mechanisms such as obesity and physical activity. In addition, previous studies have consistently reported that inflammatory cytokines and higher insulin levels increased oestrogen synthesis by aromatase activation [32, 38, 39]. Consistent with our results, a recent study [40] concluded that a more anti-inflammatory diet after breast cancer diagnosis was associated with better overall survival among postmenopausal BC survivors.

On the other hand, breast cancer hormone receptor status is considered a predictor of prognosis, and diet might have differential effects on overall survival depending on this. In stratified analysis by hormone receptor status, associations between DRRD and ISD with overall mortality were observed among PR+ subtype tumours, although interactions were not statistically significant. Owing to the high proportion of missing data on hormone receptor status we had limited power to detect differences in overall mortality between these subtypes. Indeed, we found no association with the oestrogenic dietary pattern, which is an unexpected result; we have no explanation for it.

Given the potential inter-relationship between the pathways represented by the DRRD and ISD scores, it was worth exploring the combination of DRRD and ISD on mortality among breast cancer survivors. Survivors following a low-IR and anti-inflammatory diet had a 17% lower risk of all-cause mortality compared to those following a high-IR and pro-inflammatory diet. The combination of these two dietary patterns that previously showed an individual effect on mortality is of interest to explore new dietary strategies to improve survival after breast cancer. The adjusted mean intakes (Table S7) indicated that the foods most associated with a low-IR and anti-inflammatory diet, were vegetables, legumes, fruits, nuts and seeds, yogurt, non-white bread, fish and shellfish, fruit and vegetable juices, coffee, and tea. On the contrary, higher means in the group of high-IR and a pro-inflammatory diet were seen for milk, cheese, fresh and processed meat, butter, the sugar and confectionery group, cakes, and alcoholic beverages.

Strengths of this study include its prospective design, the large number of breast cancer survivors and events (deaths), the long follow-up from the date of diagnosis, and detailed information on potential confounders. Furthermore, the availability of dietary components from which dietary patterns are derived from standardised dietary intake using means and standard deviations derived from EPIC captures the main food groups consumed by the European population.

One important limitation of our study is the lack of information on treatment, which is a strong determinant of prognosis and survival. To mitigate this issue, at least partially, we used the available information on tumour stage at diagnosis, grade of tumour differentiation and receptor status as a potential surrogate for treatment, since these characteristics often determine the therapeutic approach in these patients. In addition, we also considered the hypothesis that patients with an older diagnosis would have a worse prognosis than newly diagnosed patients due to advances in treatment. However, the effect estimates remained similar in sensitivity analyses for different time periods. Secondly, dietary intakes were measured only once at baseline, which may not be sufficient in determining the patient’s usual intake over the years and after diagnosis. Despite this, sensitivity analyses concluded that the association for high adherence to DRRD and to a lesser extent to ISD was maintained across the different time periods from diet measurement to diagnosis. Finally, as in every observational study, residual confounding is possible, although we controlled for a wide range of predictors of diet and breast cancer mortality.

In conclusion, our findings from a large prospective cohort study suggest that anti-diabetic (or low insulin resistance) and anti-inflammatory diets prior to breast cancer diagnosis are associated with mortality among breast cancer survivors. This may be tempered by the fact that we did not see a clear association with improved breast cancer outcomes; moreover chronic inflammation and hyperinsulinemia maybe be related to a variety of causes of death. Nevertheless, although we do not fully understand the pathways and mechanisms, the long-term adherence to anti-diabetic and anti-inflammatory dietary patterns could be a means to improve the prognosis of breast cancer survivors, and hence could help provide dietary recommendations. Further studies using dietary patterns related to biological mechanisms, especially nutritional intervention studies, are warranted.

## IARC disclaimer

Where authors are identified as personnel of the International Agency for Research on Cancer/World Health Organisation, the authors alone are responsible for the views expressed in this article and they do not necessarily represent the decisions, policy, or views of the International Agency for Research on Cancer/World Health Organisation.

## Supplementary information

Restricted cubic splines models of the scores and overall mortality

Specific causes of death

Descriptive of breast cancer survivors across EPIC countries

Food items included in the DRRD

Food items included in the ISD

Food items included in the ERDP

Models for different versions of DRRD score

Adjusted mean intakes of food groups for ISD and DRRD scores

## Data Availability

EPIC data are available for investigators who seek to answer important questions on health and disease in the context of research projects that are consistent with the legal and ethical standard practices of IARC/WHO and the EPIC Centres. The primary responsibility for accessing the data belongs to IARC and the EPIC centres. Access to materials from the EPIC study can be requested by contacting epic@iarc.fr.

## Code availability

All statistical analyses were performed using R 4.1.2 statistical software. Code can be made available upon reasonable request.
