# Supplementary material for: Dietary patterns related to biological mechanisms and survival after breast cancer diagnosis: results from a cohort study
Source: Br J Cancer. 2023 Feb 3;128(7):1301–10. doi: 10.1038/s41416-023-02169-2 (PMC10050013; doi:10.1038/s41416-023-02169-2)
Supplement: Supplementary file 1 — Restricted cubic splines models of the scores and overall mortality [file 41416_2023_2169_MOESM1_ESM.pdf]

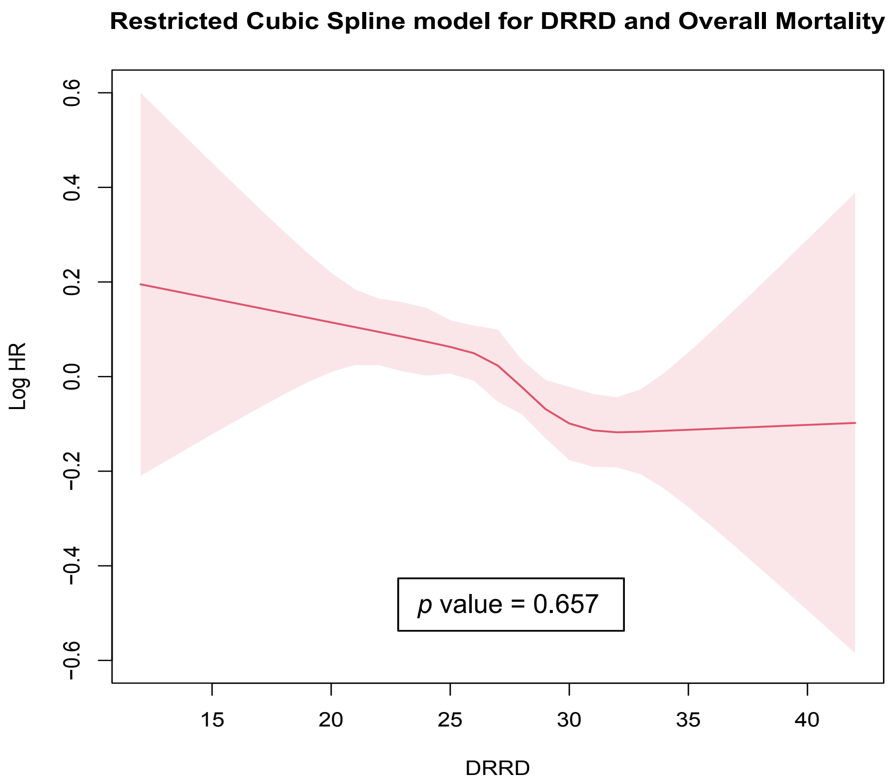

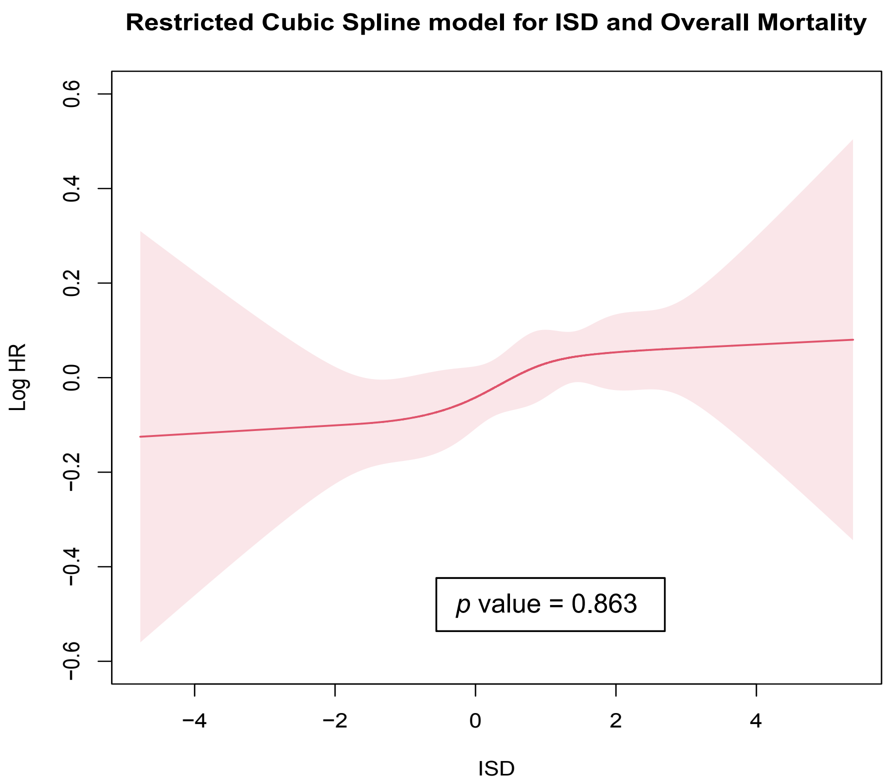

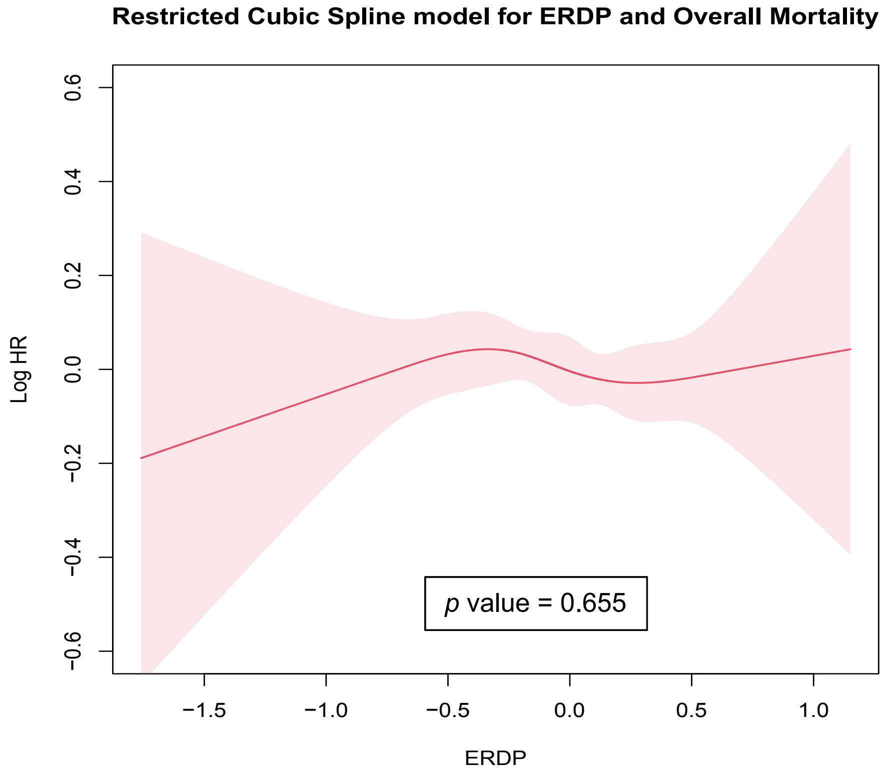

Figure S1. Restricted cubic spline models for each of the dietary patterns and overall mortality.

Restricted cubic splines with three knots placed at the 25th, 50th, 75th percentiles for each dietary pattern from multivariable adjusted models.

P-values obtained by testing for non-linearity using a likelihood ratio test comparing two multivariable nested models: one model with only a linear term and one with a linear term and restricted cubic spline terms.

Multivariable models stratified by country and menopausal status at diagnosis and adjusted for age at diagnosis (5-years categories), attained level of education, physical activity, body mass index, alcohol consumption (g/day) reported at recruitment, smoking habit and intensity as cigarettes per day (cig/d) at recruitment, ever use of hormone for menopause at diagnosis, cancer stage at diagnosis, cancer grade, and tumor receptor status: ER, PR, HER2.
